# The Role of Multimodality Imaging in Atrial Fibrillation and Heart Failure: From Patient Selection to Procedural Ablation Guidance

**DOI:** 10.3390/medicina62071296

**Published:** 2026-07-05

**Authors:** Elena Marchetti, Angelo Melpignano, Rita Pavasini, Michele Malagù, Francesco Vitali, Laura Rotondo, Maria Lo Monaco, Rocco Mollace, Gianluca Campo, Matteo Bertini, Federico Marchini

**Affiliations:** 1 Cardiology Unit, Azienda Ospedaliero Universitaria di Ferrara, Via Aldo Moro 8, 44124 Ferrara, Italy; elena02.marchetti@edu.unife.it (E.M.); angelo.melpignano@edu.unife.it (A.M.); pvsrti@unife.it (R.P.); mlgmhl1@unife.it (M.M.); vtlfnc@unife.it (F.V.); laura.rotondo@edu.unife.it (L.R.); cmpglc@unife.it (G.C.); brtmtt2@unife.it (M.B.); mrcfrc2@unife.it (F.M.); 2 Cardiovascular Institute, Ospedale Humanitas Gavazzeni, Via Mauro Gavazzeni 21, 24125 Bergamo, Italy; rocco.mollace@gavazzeni.it

**Keywords:** atrial fibrillation, heart failure, multimodality imaging, transthoracic echocardiography, trans-esophageal echocardiography, cardiovascular magnetic resonance, cardiac computed tomography

## Abstract

Atrial fibrillation (AF) and heart failure (HF) frequently coexist and mutually worsen prognosis, creating a complex clinical scenario in which treatment decisions are increasingly imaging driven. Catheter ablation has emerged as a pivotal rhythm control strategy in selected patients with HF, but careful phenotyping of the atrial and ventricular substrate is essential to balance potential benefits against procedural risk and the likelihood of durable sinus rhythm. In this narrative review, we summarize the role of multimodality imaging across the entire AF care pathway in patients with HF, from candidate selection to intraprocedural guidance and post-ablation follow-up. Ultrasound imaging remains the cornerstone of pre-procedural assessment. Cardiac computed tomography (CCT) refines anatomical characterization of the left atrium, pulmonary veins, and left atrial appendage. Cardiovascular magnetic resonance (CMR) offers comprehensive tissue characterization of atrial and ventricular fibrosis, allowing distinction between atrial primary and atrial secondary AF phenotypes and informing expectations of reverse remodelling. During ablation, intracardiac echocardiography and transesophageal echocardiography optimize transseptal access, catheter navigation, and complication monitoring, and they are particularly relevant with contemporary Pulsed Field Ablation systems. In follow-up, echocardiography, CCT, and CMR are pivotal for quantifying structural reverse remodelling and detecting rare but life-threatening complications such as atrio esophageal fistula and pulmonary vein stenosis. An integrated, multimodality, substrate-based imaging strategy is therefore crucial to personalize rhythm versus rate control decisions and to guide safe, effective ablation in patients with AF and HF.

## 1. Introduction

Atrial fibrillation (AF) and heart failure (HF) represent two of the most pervasive cardiovascular challenges of the 21st century, due to their increasing prevalence and frequent coexistence. AF is the most represented arrhythmic cardiac condition worldwide, affecting 11.7 million people in Europe and 10.5 million in America while HF affects 64 million people in the world [1,2,3].

These two conditions often occur simultaneously with their coexistence associated with increased risk of cardiac outcomes. Moreover, these two conditions share the same risk factor: advanced age, structural heart disease, hypertension, obesity and diabetes.

It is estimated that over 30% of patients with AF develop HF and, conversely, that over one-third of patients with HF experience episodes of AF [4]. This association is not merely statistical but reflects a complex pathophysiological interaction where the two conditions fuel each other: AF impairs cardiac hemodynamics through the loss of atrial contribution and RR interval irregularity, potentially leading to tachycardia-induced cardiomyopathy, while HF promotes structural and electrical remodelling of the atria, creating a favourable substrate for the maintenance of the arrhythmia [5].

Historically, the management of AF in patients with HF was based on the results of studies such as PIAF, RACE and AFFIRM, which showed no significant mortality benefits for a rhythm control strategy compared to a rate control strategy [6,7,8]. However, the advent of safer and more effective transcatheter ablation techniques has reopened the debate. Landmark clinical trials such as CASTLE-AF have demonstrated that in selected patients with heart failure with reduced ejection fraction (HFrEF), transcatheter ablation significantly reduces all-cause mortality and heart failure hospitalizations compared to standard medical therapy [9].

In this rapidly evolving scenario, the role of multi-modality imaging has transitioned from a purely diagnostic function to a cornerstone of the clinical decision-making process. The integration of echocardiography, cardiac computed tomography (CCT), and cardiac magnetic resonance (CMR) now allows not only for the characterization of anatomy but also for defining the quality of the atrial substrate (e.g., extent of fibrosis), guiding the choice between a rhythm control strategy and a rate control strategy.

While atrial remodelling directly influences the technical feasibility and success of catheter ablation, comprehensive phenotyping of the ventricular substrate also plays a paramount role in patient selection and risk stratification. In fact, characterizing the ventricular myocardium allows clinicians to differentiate between distinct heart failure phenotypes, such as heart failure with reduced ejection fraction (HFrEF) and heart failure with preserved ejection fraction (HFpEF). In HFrEF patients, identifying a tachycardia-induced cardiomyopathy substrate is critical, as these individuals derive the highest prognostic benefit from rhythm control, often showing dramatic ventricular functional recovery. Conversely, in the HFpEF population, the presence of advanced ventricular hypertrophy, diffuse interstitial fibrosis, or localized myocardial scar can profoundly influence the outcome. In fact, these ventricular alterations sustain high filling pressures and chronic diastolic stress on the atrial walls, increasing the risk of recurrence. Therefore, multimodality ventricular substrate evaluation is mandatory to anticipate both the hemodynamic response and the long-term durability of sinus rhythm [10].

This narrative review aims to analyze the role of multi-modality imaging in the management of patients with AF and HF, from diagnosis to treatment, with a specific focus on the implications for modern ablation techniques, including the promising Pulsed Field Ablation (PFA), outlining how an image-guided approach can optimize clinical outcomes and left ventricular functional recovery.

## 2. Pre-Procedural Imaging

**Figure 1:**
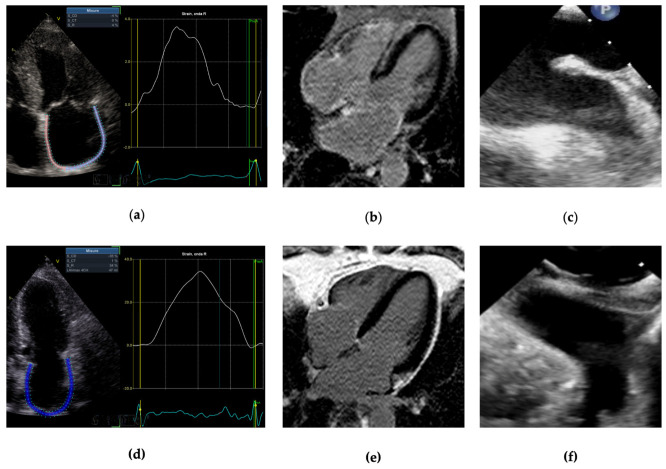
(**a**) Dilated left atrium with reduced PALS; (**b**) cardiac magnetic resonance with presence of left atrial LGE; (**c**) left atrial appendage with thrombotic formation; (**d**) Non-dilated left atrium with normal PALS; (**e**) cardiac magnetic resonance without left atrial LGE; (**f**) left atrial appendage without thrombotic formations.

Pre-procedural imaging plays a pivotal role in patients with HF and AF enabling the correct selection of the candidates for rhythm control strategies and improving procedural safety and efficacy (Table 1, Figure 1).

### 2.1. Ultrasound Imaging

Transthoracic echocardiography (TTE) represents the first imaging modality in the pre-procedural setting thanks to its wide availability and low cost. In patients with HF, TTE is fundamental to assess left ventricular diastolic and systolic function, atrial size and presence of valve diseases. Left atrial (LA) enlargement is a well-established predictor of AF recurrence following rhythm control strategies. In HF patients, LA dilation and dysfunction occur early in the onset of left ventricular (LV) diastolic dysfunction due to elevated filling pressures. TTE is preferred over transesophageal echocardiography (TOE) to assess LA size: a left atrial volume index (LAVi) greater than 34 mL/m^2^ is considered indicative of atrial structural remodelling [11]. In patients undergoing electrical cardioversion, a recent meta-analysis showed an increase in recurrence risk of around 6% for every 1 mL/m^2^ increase in LAVi, with a mean value of 40.56 mL/m^2^ observed in patients who successfully maintained sinus rhythm [12]. In a similar way, in the setting of catheter ablation, patients with markedly increased LAVi (around 48–50 mL/m^2^) show a recurrence rate of 40–50% at around 1 year after a single procedure [13]. In HF patients, TTE-derived E/e’ ratio and pulmonary artery systolic pressure (PASp) should be integrated with LAVi to differentiate between an atrial-driven process and a secondary remodelling due to chronic pressure overload.

In addition, more advanced echocardiography techniques, like atrial strain analysis (LAS), have emerged as early markers of atrial myopathy and can provide more prognostic data [14]. LAS reflects the three fundamental components of left atrial cycle: reservoir phase of expansion during ventricular systole (PALS), conduit phase during early diastole (LAScd) and contractile phase during late diastole (PACS). Among these parameters, PALS has emerged as the most robust for prediction of rhythm control. PALS reflects atrial compliance and ability to store venous return; its reduction occurs early in the disease process, often preceding atrial enlargement, thus allowing for earlier identification of subclinical atrial myopathy. Different studies have evaluated the prognostic role of PALS after catheter ablation, showing that values of PALS ≤ 18–25% are usually associated with a higher risk of AF recurrence [15]. Notably, in HF patients, PALS has demonstrated superior sensitivity in predicting the failure of rhythm control compared to traditional volumetric markers, as it directly reflects the increased atrial stiffness inherent to the HF phenotype.

TOE is routinely used before ablation or cardioversion to exclude left atrial appendage (LAA) thrombus, with a specificity, sensitivity, and accuracy of 100%, 93%, and 99%, respectively [16]. Some centres perform AF ablation without pre-procedural assessment of LAA thrombus, relying solely on uninterrupted anticoagulation; however, this strategy has limitations, as anticoagulation alone does not fully eliminate the risk of LAA thrombus, particularly in HF patients [17]. However, TOE diagnostic accuracy in LAA thrombus detection can be influenced by the presence of spontaneous echocardiographic contrast (SEC) or Sludge. SEC represents a reversible aggregation of red blood cells in a low-flow state, while Sludge is considered a more advanced stage of blood stasis, appearing as a viscid, dynamic, gel-like echogenicity that persists throughout the cardiac cycle. Both SEC and Sludge have been independently associated with increased thromboembolism even in the absence of LAA thrombi [18,19]. In the presence of SEC and/or Sludge, TOE images can be nondiagnostic due to reverberation artefacts; to overcome these situations, ultrasound-enhancing agents (UEAs) can be used to achieve a better diagnostic accuracy. TOE can also add information on LAA hemodynamic status. Beyond direct thrombus visualization, measurement of peak emptying flow velocity is a powerful functional marker, with velocities <20–25 cm/s being independently associated with an increased thromboembolic risk in the post-procedural setting, even in the absence of frank thrombotic formation in the LAA [20]. To conclude, pre-procedural TOE can also be used to evaluate the anatomy of the interatrial septum which, in the setting of transcatheter (TC) ablation, can modify the transseptal puncture approach. The presence of Patent Foramen Ovale (PFO) may in fact simplify the access to the left atrium; on the other hand, the presence of interatrial septal aneurysm (IAS) or lipomatous hypertrophy may require the use of specialized needles (i.e., radiofrequency-powered needles).

### 2.2. Cardiac Computed Tomography

**Figure 2:**
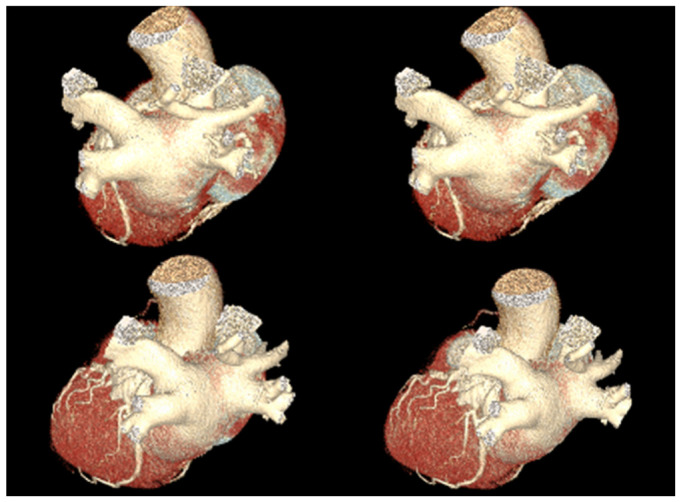
Three-dimensional volume-rendered Computed Tomography (CT) reconstruction of the left atrium and pulmonary veins.

CCT can provide additional information, especially before transcatheter ablation, offering a detailed anatomical assessment of the left atrium, pulmonary veins and LAA anatomy (Figure 2). CCT allows for the detection of any pulmonary veins’ variant (i.e., common ostia, accessory pulmonary veins) which can influence the ablation strategy and assist the operator in choosing the most suitably sized catheter and in positioning the catheter during the procedure [21]. The integration of CT imaging into electro-anatomical mapping systems has been shown to improve catheter navigation, reducing fluoroscopy time and increasing the precision of lesions delivery [22]. A recent study has also proposed a possible role of cardiac CT in the identification of atrial ablation substrate. In particular, a reduction in CT-derived left atrial wall thickness (LAWT) has been associated with the presence of arrhythmogenic low-voltage zones and extensive fibrosis, which may increase the risk of procedural failure and AF recurrence. From a procedural point of view, areas presenting a significant thinning (LAWT < 1 mm) are more prone to arrhythmia recurrence but also represent high-risk zones for esophageal fistulae or perforation. Therefore, pre-procedural LAWT mapping can allow a tailored energy delivery, leading to effective transmural lesions while minimizing the risk of collateral damage and perforation [23]. CCT can also be useful in the identification of the right pericardiophrenic artery, which serves as an anatomical landmark for the right phrenic nerve; this could help to identify patients at risk of phrenic nerve injury during the ablation procedure [24]. CCT has also emerged as an important tool to identify the presence of LAA thrombus, especially when TOE carries elevated risk (e.g., difficult intubation, esophageal pathology). In this setting, delayed-phase imaging is essential to exclude thrombus, with a second data acquisition following a short delay of 30 s to 180 s (ideally 60 s) from the initial contrast peak. The importance of delayed-phase imaging is to differentiate true thrombus from slow flow, which is common in the setting of AF and HF and is equivalent to SEC/Sludge seen on TOE [25]. Specifically, in a meta-analysis, CCT has shown a sensitivity of 96%, specificity of 92%, and overall accuracy of 94%; while when delayed imaging was used, sensitivity increased to 100%, specificity to 99%, and accuracy to 99% [26].

### 2.3. Cardiac Magnetic Resonance

CMR, similarly to CCT, is effective in assessing the presence of LAA thrombus, with long inversion time (600 ms)-delayed enhancement acquisition providing the best results, achieving 100.0% sensitivity, 99.0% specificity, and 99.38% accuracy [27]. In addition, CMR allows for tissue characterization through myocardial and LA late gadolinium enhancement (LGE), extracellular volume (ECV) and T1 mapping, enabling the quantification of atrial and ventricular fibrosis. Higher values of native T1 mapping of the LV have been shown to be independently associated with higher risk of recurrence in patients with paroxysmal AF [28]. Similarly, the incidental finding of left ventricle LGE [29], and increased ECV [30], have been shown to be independently associated with AF recurrence post-ablation. Beyond ventricular assessment, LGE can also evaluate LA fibrosis, typically categorized using the Utah Classification. In the DECAAF I study, patients with extensive atrial fibrosis (Utah Stage III/IV, ≥20%) showed poor response to catheter ablation, making LA-LGE an important parameter for selecting candidates for rhythm control [31]. CMR-derived data on LA fibrosis can be integrated into electro-anatomical mapping to guide AF ablation; however, this strategy did not show benefit in terms of rhythm outcome in patients with persistent AF in the DECAAF II study [32]. In HF patients, this can help the clinician to distinguish between an atrial-primary AF, where rhythm control may result in long-term outcomes, and an atrial-secondary AF, where advanced myocardial and atrial fibrosis (identified via CMR-LGE) may suggest that a rate control strategy is a more realistic and safer therapeutic goal. Furthermore, similarly to STE, CMR-Feature Tracking (FT) has recently emerged as a robust imaging tool to evaluate atrial deformation, overcoming problems related to bad acoustic windows [33].

Both CCT and CMR are also routinely used in clinical practice for the identification of epicardial adipose tissue (EAT), whose amount has been shown to be associated with high risk of AF recurrence post-ablation, especially if located around the left atrium [34,35]. This is particularly relevant in the heart failure with preserved ejection fraction (HFpEF) population, where EAT-driven inflammation is a primary driver of the atrial-ventricular disease continuum [36].

The integration of echocardiography, CT and CMR shifts the pre-procedural assessment from a purely anatomical evaluation to a comprehensive substrate-based approach.

## 3. Intra-Procedural Imaging

### 3.1. Ultrasound Imaging for Atrial Fibrillation

Ultrasound imaging guidance during interventional procedures decreases fluoroscopy radiation dose and improves anatomic visualization; therefore, its application is considered essential throughout all phases of the procedure [37].

### 3.2. TOE

The primary role of intraprocedural TOE during radiofrequency ablation for AF is to provide real-time guidance for transseptal puncture and anatomical visualization of the left atrium and pulmonary veins. TOE assists in identifying the optimal transseptal access site, monitoring for complications such as pericardial effusion, and confirming catheter position relative to critical structures, particularly in patients with complex anatomy or prior cardiac surgery [38,39,40,41,42,43,44].

Additionally, TOE can be used for immediate assessment of thrombus formation in the left atrium or left atrial appendage during the procedure, though this is performed less frequently than pre-procedural screening. In selected cases, TOE may aid in mapping and ablation by visualizing pulmonary vein ostia and branches, potentially reducing dependence on angiography and fluoroscopy [43], with procedural success and arrhythmia recurrence rates similar to those achieved with intracardiac echocardiography (ICE). However, according to the American College of Radiology, TOE has limitations in evaluating detailed pulmonary venous anatomy compared to other imaging modalities such as ICE or cardiac CT [45]. TOE is typically performed under sedation and may increase both procedure duration and complexity. It is most valuable in patients with challenging anatomy, elevated risk of complications, or when ICE is unavailable or contraindicated [43,44].

### 3.3. ICE

ICE offers superior procedural safety and efficiency compared to TOE for real-time guidance during atrial fibrillation ablation, with equivalent effectiveness in achieving arrhythmia outcomes and preventing thromboembolic events [46,47,48,49]. ICE is associated with fewer overall complications—including cardiac tamponade and mortality—as well as reduced fluoroscopy time, radiation exposure, and contrast use [48]. It enhances procedural workflow by providing continuous anatomical visualization, guiding transseptal puncture, and allowing immediate detection of complications such as pericardial effusion or thrombus. The use of ICE increases the likelihood of first-pass pulmonary vein isolation and successful isolation of all veins, while reducing the need for repeat ablations [46,47,48]. Moreover, ICE allows for real-time, operator-controlled visualization of cardiac structures for precise cryoballoon positioning at the pulmonary vein ostia and for assessment of venous occlusion, reducing contrast agent use and enhancing procedural efficiency [50,51,52].

### 3.4. TOE vs. ICE

The most recent and robust evidence directly comparing ICE and TOE for intraprocedural guidance during atrial fibrillation ablation stems from a 2025 multicentre randomized clinical trial, supported by contemporary meta-analyses and large cohort studies [48,53,54].

The 2025 randomized trial by Hu et al. established that ICE is non-inferior to TOE for pre-procedural thrombus detection and the prevention of periprocedural thromboembolic events, supporting its use as a viable alternative in clinical practice [54].

Confirming these findings, meta-analyses report that ICE-guided ablation is associated with lower odds of procedural complications (including cardiac tamponade and mortality), shorter procedure and fluoroscopy times, reduced radiation exposure, and higher rates of first-pass pulmonary vein isolation—all while achieving similar long-term arrhythmia recurrence rates [48,50].

ICE is now considered a valid alternative to TOE for intraprocedural guidance, especially when real-time anatomical visualization, rapid complication detection, or patient intolerance to TOE are considered. TOE retains its role as the established standard for pre-procedural thrombus exclusion, particularly in settings with limited ICE resources or expertise, as endorsed by the Heart Rhythm Society consensus [53].

**Figure 3:**
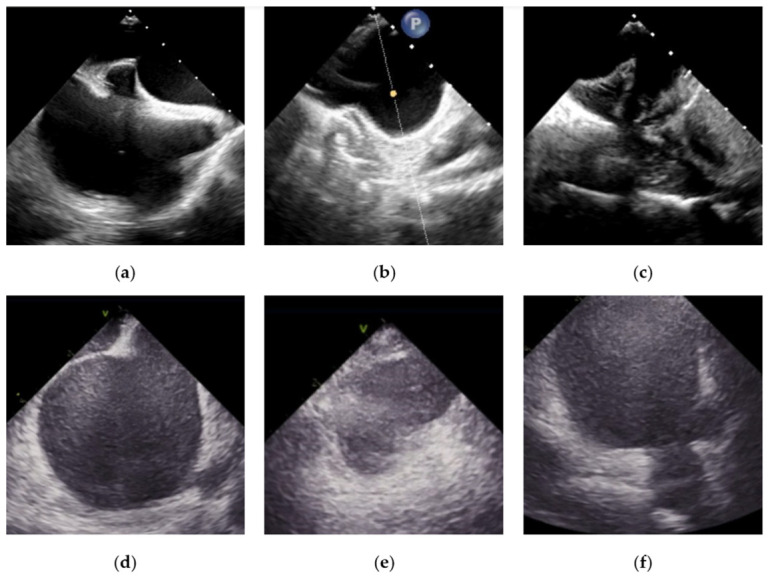
(**a**) Transseptal puncture TEE-guided; (**b**) Right pulmonary veins on TEE view; (**c**) Left pulmonary vein on TEE view; (**d**) Transseptal puncture ICE-guided; (**e**) Right pulmonary veins on ICE view; (**f**) Left pulmonary veins on ICE view.

Regarding procedural safety, ICE provides real-time visualization of the interatrial septum, ensuring a highly secure TSP by directly monitoring the needle tenting on the fossa ovalis and ruling out inadvertent aortic root or posterior wall staining. However, a notable technical limitation of ICE compared to TOE is the lack of simultaneous orthogonal views. While TOE easily allows for biplanar imaging (combining short-axis and bicaval views) to precisely confirm the infero-posterior spatial orientation of the puncture site, standard ICE mandates active catheter manipulation—such as clockwise rotation and posterior deflection—to reconstruct the septal anatomy [55]. Despite the lack of an orthogonal plane, the immediate feedback on mechanical tenting and the ability to combine ICE with electroanatomical mapping (EAM) effectively overcome this limitation, maintaining an optimal safety profile and a minimal risk of inadvertent punctures (Figure 3).

### 3.5. Pulsed Field Ablation of Atrial Fibrillation

Recently, Pulsed Field Ablation (PFA) has emerged as a novel technique for atrial fibrillation ablation, through electroporation. This mechanism allows for tissue selectivity targeting the myocardium, thereby reducing the risk of damage to adjacent structures such as the esophagus, phrenic nerve, and pulmonary veins [56,57,58,59]. Multicentre studies and randomized trials (ADVENT, AdmIRE, SINGLE SHOT CHAMPION) have demonstrated that PFA is at least non-inferior to thermal techniques (radiofrequency, cryoballoon) in terms of efficacy, with 12-month freedom from arrhythmia recurrence rates between 73% and 75% [57,59,60,61,62].

Regarding intraprocedural imaging, both ICE and TOE are used to guide transseptal puncture, catheter positioning, and complication monitoring. ICE offers advantages in terms of direct visualization, reduced risk of complications, and the ability to perform the procedure with minimal or no fluoroscopy, especially with PFA systems integrated with electroanatomic mapping [60,63]. Recently, TOE-guided PFA has emerged as an interesting imaging strategy to guide PFA. While robust data remain limited, initial case series have reported high procedural success, with first-pass isolation in all veins and no major complications. Compared to ICE, TOE offers a substantially lower cost profile and equivalent imaging resolution when performed under general anesthesia [64].

### 3.6. Non-Ultrasound Imaging for Atrial Fibrillation Ablation

The most used non-US intraprocedural imaging techniques in atrial fibrillation ablation—using either radiofrequency, cryoballoon or Pulsed Field Ablation—are CCT and CMR. These techniques are not employed in real-time during the procedure; instead, their anatomical data are integrated into electroanatomic mapping systems for navigation and lesion planning [65,66].

Cardiac CT is the most widespread method for three-dimensional reconstruction of atrial and pulmonary vein anatomy, facilitating procedural planning and fusion with mapping systems [21,65,67]. CT integration reduces fluoroscopy times and improves catheter positioning accuracy for all energy types (radiofrequency, cryoballoon, PFA) [22].

CMR, particularly with LGE, allows for the assessment of pre-procedural atrial fibrosis and the visualization of post-ablation lesions. CMR can be integrated into mapping systems to guide the ablation strategy, especially in radiofrequency and PFA, where lesion characterization and fibrosis assessment are crucial [53,68,69,70,71].

Pre-procedural cardiac CT and CMR are usually imported into the electroanatomic mapping system as segmented three-dimensional datasets and registered to the live procedural anatomy using landmark-based or surface-based alignment. In this way, CT provides detailed left atrial, pulmonary vein, and left atrial appendage anatomy, whereas CMR adds substrate information such as atrial fibrosis; the fused model can then support transseptal puncture, catheter navigation, and lesion planning, often in combination with intracardiac or transesophageal echocardiography for real-time confirmation.

Currently, the direct, real-time use of CT or CMR during the procedure is limited by technical complexity and the availability of dedicated systems; their primary application is in the planning phase and for navigation via fusion with electroanatomic mapping [53,65,66]. The Heart Rhythm Society notes that real-time CMR is still under development and is not part of routine clinical practice [53].

Electroanatomical mapping systems (such as CARTO, Rhythmia, or EnSite) represent the central platform for non-fluoroscopic catheter navigation and substrate characterization during AF ablation. By tracking catheter electrodes within a magnetic or impedance-based field, all EAM systems generate real-time, high-resolution three-dimensional shells of the left atrium. These systems allow operators to precisely correlate electrical information with cardiac anatomy, which is essential for identifying arrhythmogenic substrates and targeting low-voltage fibrotic areas. Furthermore, the capacity of EAM to undergo image fusion with pre-procedural CMR or CT datasets maximizes lesion delivery accuracy while minimizing operator reliance on ionizing radiation [72,73].

Building on this technological integration, contemporary clinical workflows highlight the feasibility of entirely “zero-fluoroscopy” approaches. The systematic reliance on high-density EAM may enable operators to perform the entire procedure without any radiation exposure, completely eliminating radiological risks for both patients and laboratory staff [74].

## 4. Imaging During Follow-Up

In the management of AF associated with HF, follow-up imaging is essential to monitor the reverse remodelling of the left ventricle and left atrium, to evaluate the success of the chosen strategy, to detect any subclinical progression of myocardial dysfunction and to promptly assess any procedural complications.

### 4.1. Reverse Remodelling

TTE represents the cornerstone of long-term assessment for patients with AF and HF, regardless of the chosen management strategy. The primary objective of TTE during follow-up is to document signs of structural reverse remodelling, which is the most significant indicator of therapeutic success. Despite advanced baseline remodelling, reverse structural remodelling remains more likely in patients with smaller left atrial size, less extensive atrial fibrosis, preserved left atrial strain, and better left ventricular function, particularly when AF duration is shorter and the arrhythmia is paroxysmal rather than persistent [75]. Similarly, decreases in left ventricular end-diastolic and end-systolic volumes serve as objective markers of improved cardiac efficiency. In patients without evident improvement in systolic function or LV volumes, systematic assessment of LV Global Longitudinal Strain (GLS) can identify subtle myocardial improvements and monitor recovery. In patients with suspected tachycardia-induced cardiomyopathy (TCM), assessment of left ventricle systolic function is fundamental to guide further therapeutic interventions. In these patients, persistent dilation of the LV and LA can be seen even after restoration of sinus rhythm, making them at high risk of arrhythmia recurrence and further episodes of TCM, thus requiring adequate pharmacological treatment [76]. Post-procedural LA size and remodelling are other important parameters to be evaluated, especially in patients with HF, since post-procedural LA structural changes seem to predict AF recurrence [77]. Successful AF ablation is usually associated with reduction in LA dimensions and improvement in LA function; however, this reduction seems to be related to the time of onset of AF: in persistent and long-standing persistent AF, LA dimensions significantly decrease within one year after ablation, while in patients with paroxysmal AF, no significant structural changes are seen [78]. A recent study has shown that in patients with persistent AF, LAVi decreases approximately 10 mL/m^2^ after ablation and that the magnitude of LA volume reduction correlates with better outcomes [79].

### 4.2. Procedural Complications

CCT and CMR are usually used in the post-ablation setting to detect any relevant complication. Although rare, the most common complications in TC ablation include esophageal injury and pulmonary vein stenosis (PVS). Atrio-esophageal fistula (AEF) occurs in ≤0.2% of ablation procedures; however, it carries an elevated mortality (up to 60–80%); hence, rapid recognition and treatment are fundamental [80]. It usually presents 2–6 weeks after the index procedure and major symptoms include fever, chest pain, odynophagia, and neurological deficits [81]. It commonly arises following posterior left atrial wall ablation since, in the vast majority of the population, the esophagus lies adjacent to the left pulmonary veins separated from the left superior pulmonary vein (LSPV) by a mean distance of 10 mm and from the left inferior pulmonary vein (LIPV) by only 3 mm [82]. Chest CT, with intravenous or oral water-soluble contrast, is the preferred diagnostic tool for detection of AEF and to make differential diagnosis with pericardial-esophageal fistulas and esophageal perforation. Major chest CT findings of AEF are the presence of gas within the left atrium [21]. Chest MRI can be used alternatively, although chest CT remains the diagnostic option of choice [83]. Since neurologic symptoms are common in patients with AEF, early brain imaging is suggested to assess the severity of brain damage [80].

PVS has a reported incidence of 0.29% and in severe cases it can lead to complete occlusion of the vein with subsequent venous infarction and secondary pulmonary hypertension [83]. PVS severity is graded based on the degree of lumen narrowing into mild (<50%), moderate (50–70%), and severe (>70%) [53]. PVS usually remains asymptomatic until the vein dimension is reduced significantly (>50% stenosis) or lung perfusion is decreased by >20–25% [84]. Clinical manifestations usually appear 3 to 6 months after the procedure and their severity is related to the number of pulmonary veins affected. Most common symptoms include dyspnoea, cough, chest pain and hemoptysis. Imaging modalities most used in the diagnosis of PV stenosis include CT and MR angiography [81]. Routine screening for PV stenosis following ablation is no longer recommended due to the reduced risk of PVS with novel ablation technologies [53]. CT scan readily identifies the location, the length and the severity of the stenosis. During CT assessment of PV, it is important to verify ostial dimension variation during the cardiac cycle and compare it with pre-procedural images (if available) at the same time of cardiac cycle since ostial diameter may vary by up to 32% [85]. Treatment options for pulmonary vein stenosis after ablation include stenting and balloon dilatation [86,87]. TOE can also be effective in the detection of post-ablation PVS, even though CT and MR angiographies are usually preferred due to their non-invasive nature. In PVS, aliasing is usually seen at Colour Doppler, reflecting the flow turbulence associated with the stenosis. In addition, pulsed-wave Doppler can show high peak systolic and diastolic velocities (>100 cm/s) [88].

### 4.3. Scar Assessment

CMR in the post-procedural setting can be employed for lesions follow-up and to identify the presence of any residual gaps and/or arrhythmic substrates [68,69]. CMR can in fact quantify the extent and distribution of LA scar burden through LGE sequences [89,90]. As previously reported, different studies have established a direct correlation between the degree of pre-existing atrial fibrosis and the likelihood of post-procedural recurrence; however, CMR can also be useful in the post-procedural setting to verify the quality of the ablation lesions [89,91]. Specifically, the identification of gaps in the circumferential lesions around the PVs allows for the detection of potential triggers for procedural failure. Furthermore, the regional distribution of the induced scar has significant prognostic value: successful scar formation localized to the left atrial posterior wall and the septal wall has been shown to be independently associated with lower AF recurrence rates, suggesting that these areas could be critical nodes in the maintenance of the arrhythmic substrate [92].

However, the quality of CMR images is often suboptimal for scar assessment due to suboptimal rhythm control in patients with AF and the thin wall of the LA, making this technique difficult to apply in clinical practice.

## 5. Conclusions and Future Perspective

In the management of the complex relationship between AF and HF, multimodality imaging has evolved from a purely diagnostic tool into one of the major determinants of the clinical decisional process (Table 2). The integration of different imaging modalities enables a personalized approach which is essential in this high-risk population.

Pre-procedural assessment through TTE strain analysis and CMR-LGE allows for a characterization of the arrhythmic substrate, shifting the focus from simple anatomical changes to the quantification of underlying myopathy and fibrosis. This allows for selection and identification of candidates who will truly benefit from rhythm control strategies.

Intra-procedural imaging, dominated by the widespread use of ICE and TOE, has fundamentally enhanced the safety and efficacy of complex ablation maneuvers such as transseptal puncture and catheter navigation. In addition, the emerging role of fusion imaging can further minimize radiation exposure and procedural complications. Finally, post-procedural imaging remains of paramount importance for monitoring structural and functional reverse remodelling and for the early detection of rare but life-threatening complications, such as atrio-esophageal fistulas or pulmonary vein stenosis.

In conclusion, an integrated, image-guided workflow is no longer optional but necessary to navigate the challenges of AF and HF. Future advancements in artificial intelligence and automated image integration are expected to further refine these strategies, ensuring that the treatment is tailored not only to the arrhythmia but to the unique myocardial and atrial characteristics of each patient.

## Figures and Tables

**Table 1:** Multimodality imaging features for clinical stratification, strategy selection, and red flags against radiofrequency catheter ablation in patients with heart failure and atrial fibrillation.

Imaging Modality	Favour Rhythm Control	Favour Rate Control	Against AF Ablation
TTE	- LAVi ≤ 34 mL/m^2^ - PALS > 25% - No advanced diastolic dysfunction (normal E/e’, normal PASP)	- Elevated E/e’ and increased PASP (suggest secondary remodelling due to chronic pressure overload)	- LAVi > 48–50 mL/m^2^ (40–50% recurrence at 1 year) - PALS ≤ 18–25% (subclinical atrial fibrosis, high recurrence risk)
TOE	- Absence of thrombus/Sludge/SEC in left atrial appendage - LAA emptying velocity > 25 cm/s	- Reduced LAA emptying velocity (mild blood stasis)	- Persistent severe SEC or Sludge (even without thrombus) - LAA emptying velocity < 20–25 cm/s (high post-procedural thromboembolic risk)
CCT	- Standard pulmonary vein anatomy (no common ostia or accessory veins) - LAWT > 1 mm (lower risk of diffuse fibrosis and perforation) - No thrombus on delayed-phase imaging	- Anatomy variant without critical technical limitations	- LAWT < 1 mm (thinning associated with low-voltage zones and fibrosis → high recurrence and fistula risk) - Complex pulmonary vein variants (technical difficulty) - Evidence of thrombus on delayed-phase imaging
CMR	- No or mild atrial fibrosis (<20% of LA wall–Utah stage I/II) - Normal left ventricular native T1 time - No LV late gadolinium enhancement (LGE) and normal ECV	- Moderate atrial fibrosis (Utah stage II/III) - Large amount of peri-atrial epicardial fat (inflammation, especially in HFpEF)	- Extensive atrial fibrosis (≥20%–Utah stage III/IV) → poor response to ablation - Increased native T1 time, elevated ECV, or LV LGE (diffuse myocardial remodelling)

AF: atrial fibrillation; ECV, extracellular volume; HFpEF, heart failure with preserved ejection fraction; LA, left atrium; LAA, left atrial appendage; LAVi, left atrial volume index; LAWT, left atrial wall thickness; LGE, late gadolinium enhancement; LV, left ventricle; PALS, peak atrial longitudinal strain; PASP, pulmonary artery systolic pressure; SEC, spontaneous echo-contrast; TTE, transthoracic echocardiography; TOE, transesophageal echocardiography; CCT, cardiac computed tomography; CMR, cardiac magnetic resonance.

**Table 2:** Multimodality imaging in patients with atrial fibrillation and heart failure: clinical applications by phase of management.

Imaging Modality	Pre-Procedural Setting	Intra-Procedural Setting	Post-Procedural Setting
TTE	Baseline LVEF, LAVi, valvular status, and PALS for rhythm control candidate selection.	Not typically used (limited access to the patient).	Monitor of reverse remodelling (LVEF improvement, LAVi reduction, GLS recovery).
TOE	Gold standard for LAA thrombus exclusion and hemodynamic assessment (LAA flow velocities).	Guidance for TSP; monitoring for pericardial effusion.	Detection of PVS (aliasing/high velocities) and LAA thrombus if AF recurs.
ICE	Not applicable.	Real-time guidance for TSP and catheter contact; early detection of tamponade/thrombus.	Not applicable.
CCT	Detailed PV and LAA anatomy; LA wall thickness mapping; thrombus exclusion	Image fusion with EAM to guide navigation.	Diagnosis of PV Stenosis and detection of atrio-esophageal fistula.
CMR	Quantification of atrial/ventricular fibrosis; assessment of arrhythmic substrate	Real-time CMR (experimental/limited); EAM integration for lesion planning.	Assessment of scar burden, detection of gaps in ablation lines, and PVS.

Summary of the main applications of TTE, TOE, ICE, CCT, and CMR in patients with AF and HF, organized by the three phases of clinical management: pre-procedural, intra-procedural, and post-procedural. AF, atrial fibrillation; CCT, cardiac computed tomography; CMR, cardiac magnetic resonance; EAM, electroanatomical mapping; GLS, Global Longitudinal Strain; ICE, intracardiac echocardiography; LA, left atrium; LAA, left atrial appendage; LAVi, left atrial volume index; LGE, late gadolinium enhancement; LVEF, left ventricular ejection fraction; PALS, peak atrial longitudinal strain; PVs, pulmonary veins; PVS, pulmonary vein stenosis; TOE, transesophageal echocardiography; TSP, transseptal puncture; TTE, transthoracic echocardiography.

## Data Availability

All data relevant to the study are included in the article.

## Abbreviations

The following abbreviations are used in this manuscript:

AF	atrial fibrillation
HF	heart failure
CCT	cardiac computed tomography
CMR	cardiovascular magnetic resonance
HFrEF	heart failure with reduced ejection fraction
TTE	transthoracic echocardiography
LV	left ventricular
LA	left atrial
TOE	rtansesophageal echocardiography
LAVi	left atrial volume index
PASP	pulmonary artery systolic pressure
LAS	atrial strain analysis
PALS	peak atrial longitudinal strain
LAScd	atrial strain analysis during conduit phase
PACS	peak atrial contractile strain
SEC	spontaneous echocardiographic contrast
UEAs	ultrasound-enhancing agents
PFO	Patent Foramen Ovale
IAS	interatrial septal aneurysm
LAWT	left atrial wall thickness
LGE	late gadolinium enhancement
ECV	extracellular volume
FT	Feature Tracking
EAT	epicardial adipose tissue
ICE	intracardiac echocardiography
PFA	Pulsed Field Ablation
TCM	tachycardia-induced cardiomyopathy
GLS	Global Longitudinal Strain
AEF	atrio-esophageal fistula
PVS	pulmonary vein stenosis
